# New Perspectives and Strategies for the Management of Hypertension

**DOI:** 10.3390/jcdd10080346

**Published:** 2023-08-14

**Authors:** Fabio Angeli

**Affiliations:** 1 Department of Medicine and Technological Innovation (DiMIT), University of Insubria, 21100 Varese, Italy; fabio.angeli@uninsubria.it; 2 Department of Medicine and Cardiopulmonary Rehabilitation, Istituti Clinici Scientifici Maugeri IRCCS, 21049 Tradate, Italy

Hypertension is the leading preventable risk factor for cardiovascular disease and all-cause mortality worldwide [1]. Moreover, the global prevalence of hypertension remains high [2], and treatment of high blood pressure (BP) is the most common reason for the prescription of chronic drugs and for office visits [3,4].

According to epidemiological information provided by the United States National Health and Nutrition Examination Surveys (NHANES), the prevalence of hypertension in the United States is about 30% [3,4]. However, applying the new definition of hypertension recommended by the American College of Cardiology/American Heart Association (ACC/AHA, BP ≥ 140 mmHg systolic or ≥90 mmHg diastolic, or taking antihypertensive medication) the prevalence of hypertension among adults in the United States was 47% from 1999 to 2000, 41.7% percent from 2013 to 2014, and 45.4% from 2017 to 2018 [4]. The global prevalence of hypertension is similar to that in the United States, although it varies by country [2].

Despite such impressive prevalence, the last few years have been characterized by a notable paucity of innovative studies, and the proportion of treated hypertensive patients with “controlled hypertension” remains very low worldwide. Specifically, it has recently been estimated that such a proportion approaches 23% in women and 18% in men [5].

This Special Issue “Recent Advances in the Treatment of Hypertension” collects articles from the Americas, Africa, Asia, Australia, and Europe discussing several pertinent issues in this area of research, on topics spanning pathophysiology, risk stratification, control and management of hypertension.

Humberto Badillo-Alonso and coworkers report results from a randomized clinical trial comparing the effectiveness of the combination of enalapril and nifedipine for the treatment of hypertension versus empirical treatment [6]. They demonstrated that combined treatment was 31% more efficacious than conventional empirical treatment, which yielded an incremental clinical utility of 18% with high tolerability among patients in primary care [6].

A cross-section investigation analyzing data from the South African arm of the Prospective Urban and Rural Epidemiology (PURE–SA) study reported an H-type hypertension (hypertension associated with homocysteine levels ≥ 10 µmol/L) prevalence of 23% among all participants and a 45% prevalence among those with hypertension in a relatively large sample of Black South Africans recruited from both rural and urban communities [7].

A retrospective, single-center cohort study including outpatients with cardiovascular disease risk factors but without known cardiovascular disease evaluated the prognostic impact of cardio-ankle vascular index (CAVI) [8]. Importantly, results showed that CAVI improved the prediction of cardiovascular events (the addition of CAVI to a conventional risk score for coronary heart disease in Japan significantly improved the C statics from 0.642 to 0.713; *p* = 0.04) [8].

Anil T John and co-workers present the results of a 5-week randomized–controlled trial evaluating the effectiveness of high-intensity interval training (HIIT) and continuous moderate-intensity training (CMT) on BP of physically inactive pre-hypertensive young adults [9]. Both HIIT and CMT decreased BP; however, HIIT yielded more beneficial results in terms of reducing all the components of BP (systolic, diastolic, and mean arterial pressure) [9].

Two investigations from Italy analyze the role of hypertension in the era of the severe acute respiratory syndrome coronavirus 2 (SARS-CoV-2) pandemic [10]. An analysis of a pre-designed registry of patients hospitalized for coronavirus disease 2019 (COVID-19) with subsequent prospective collection of data demonstrated that exposure to angiotensin receptor blockers reduced mortality in 566 hypertensive patients hospitalized for COVID-19 [11,12]. After the evidence of a BP increase during the acute phase of SARS-CoV-2 infection [13], a systematic review and meta-analysis (including 357,387 subjects) also evaluated an increase in BP after COVID-19 vaccination as a potential adverse reaction [14,15]. Pooled results showed that the proportion of abnormal/increased BP after vaccination was 3.20% (95% CI: 1.62–6.21), and that proportions of cases of stage III hypertension or hypertensive urgencies and emergencies was 0.6% (95% CI: 0.1% to 5.1%) [15].

Finally, five review articles are included in this Special Issue. They discuss the role of artificial intelligence [16], the prognostic value of a tight BP control in chronic kidney disease [17], implications of endothelial dysfunction as therapeutic target [18] in hypertension and other conditions [19], the link between hypertension and cardiac arrhythmias [20,21], and recent advances in the management of hypertensive patients with a potential clinical role in the years to come (including renal denervation) [22].

In summary, to curb the detrimental impact of hypertension and its increase in prevalence worldwide, we need significant progress from a combination of new strategies, education, and technology [22]. This Special Issue includes reports gathering new insights and re-evaluating pre-existing evidence and strategies to improve the management, control, and risk stratification of hypertension.
